# Teachers’ Perspectives on Improving Online Seminars in Pharmacology: A Quantitative and Qualitative Study on Lessons Learned During the COVID-19 Pandemic

**DOI:** 10.1007/s40670-022-01634-6

**Published:** 2022-09-13

**Authors:** Ozgu Aydogdu, Michael Winder

**Affiliations:** Department of Pharmacology, Institute of Neuroscience and Physiology, the Sahlgrenska Academy, University of Gothenburg, Gothenburg, Sweden

**Keywords:** Online seminar, Pharmacology, Teachers’ perspectives, COVID-19 pandemic

## Abstract

The aim of this study is to evaluate teachers' perceptions of online seminars during COVID-19 pandemic to improve future courses in pharmacology. The study was performed as a questionnaire survey. A questionnaire that included 11 questions was used. A total of 14 online seminar teachers, of which 9 were senior teachers and 5 were PhD students, filled out the questionnaire. PhD students’ and senior teachers’ answers to questions 1–5 were compared statistically. The results of questions 6–10 were analysed qualitatively through thematic content analysis. There were no significant differences between senior teachers and PhD students in regard to the scores given to questions 1–5 in the questionnaire. Most (65%) teachers scored the online seminars lower than in person seminars. Interaction, communication, and group dynamics were mostly perceived to be less effective at online seminars compared to in person seminars. The main advantages of online seminars were time saving and flexibility. The main disadvantages of online seminars were reduced student interest, risk of monologue discussion and poorer communication without body language. Most teachers experienced minor technical problems with internet connection and sound quality. The teachers mentioned that better group dynamics, smaller groups, better chat functionality and clearer guidelines could help to improve online seminars. As an alternative to online seminars, blended-learning could be used. This way, one could appreciate both the richness of interactions in a face-to-face environment as well as the flexibility and convenience of online learning. Further studies comparing blended-learning and online teaching at seminars are needed to investigate this issue.

## Introduction

Seminars are a prominent feature of university education and a way to encourage active student participation [1, 2]. In online settings, seminars bear an even greater importance [3]. An online seminar differs from an in person seminar in several ways. One difference is an inability to perceive body language during online seminars, which can have a negative effect on communication [3, 4]. Creating a group identity and social interactions can also be more difficult at online seminars than in person seminars. In addition, online seminars require the facilitators to have technical proficiencies that are not required in face-to-face meetings [3, 5].

As evident by the transition to online education during the COVID-19 pandemic and with the latest technological improvements, new methods for university education have led to altered ideas about learning [6]. Educational methods such as online seminars reject the theories that regard knowledge as stored in students’ minds and learning as the individual acquisition of knowledge [1, 7]. Instead, the idea that learning in higher education is a social and participatory process has gained traction. During online seminars, students have an increased opportunity to develop their communication skills and critical thinking [1, 4]. Meanwhile, the importance of a good teacher is maintained as the teacher designs the seminar, assigns individual students different tasks, keeps the discussion focused and ensures that all students participate. Teachers should be aware of their responsibilities and potential barriers like technical problems during online seminars [8, 9].

It is possible that face-to-face interaction with students is required to optimize teaching and discussion during seminars. In addition, there is a risk that a teacher may not be able to fulfil all necessary tasks during online seminars. The design sometimes needs to be different during online seminars, as compared to in person seminars, which requires other and sometimes more advanced teacher skills [5].

The Department of Pharmacology at the University of Gothenburg, Sweden, is responsible for several courses that include teaching seminars. Based on the learning objectives, seminars have an extremely important function when it comes to student-activating teaching. Prior to the COVID-19 pandemic, all seminars were given at in person. However, because of restrictions, all seminars were performed online during the COVID-19 pandemic. In the current study, we aimed to investigate pharmacology teachers’ perspectives on improving online seminars.

### Differences Between Online Learning and Classroom Learning in General

Bernard et al. defined that distance education/online learning refers to teaching/learning conditions where students and teachers are physically separated from each other but have two-way communication that connects them [10]. They further concluded that distance education/online learning can be carried out synchronously or asynchronously. It has widely been discussed how distance education/online learning is different from classroom learning. Bernard et al. mentioned that there was a wide variability among studies that compared distance education/online learning and classroom learning and there was little difference between these two teaching/learning patterns [10]. In a meta-analysis of three different types of interaction teaching (student–student, student–teacher and student-content) in distance education, the authors noted that the distance that separated the activities of teaching/learning and the media that were essential for distance education were among the most cited differences [11]. In a previous meta-analysis, distance education was compared to classroom learning in terms of achievement, attitudes and course completion [12]. The authors divided the studies into asynchronous and synchronous distance education and reported different results for these two types of distance education methods. According to the findings of this meta-analysis, asynchronous distance education was more positive than classroom learning in terms of achievement and attitudes compared to synchronous distance education but had a bigger problem with course completion. It was previously mentioned that distance education could be both better and worse compared to classroom learning based on measured educational outcomes and that some pedagogical features of distance education could be associated with increased student achievement [11]. In a previous meta-analysis and review of online learning studies, Means et al. aimed to address the effectiveness of online learning, compared with classroom learning [13]. The authors concluded that online learning was as effective as classroom learning. According to the findings from prior meta-analyses, the authors also mentioned that distance education/online learning can successfully replace classroom learning when it is the only option. In another meta-analysis of job-related courses, Sitzmann et al. compared online learning and classroom learning [14]. The authors found better declarative knowledge outcomes with online learning compared to classroom learning. Procedural learning was similar in both teaching/learning settings.

### Planned Online Learning Versus Emergency Remote Instruction

Planned online learning is meaningfully different from emergency remote instruction [15]. Several reports have explored the importance of realizing the difference between planned online learning activities and emergency remote instruction [16–18]. Emergency remote instruction that can be used in a disaster, or a pandemic like COVID-19, can be a necessary substitute for classroom learning. However, planned online learning is always considered the superior teaching pattern [16]. Planning, preparation and development time for a planned online university course is approximately 6 months [15]. However, this time is much shorter for emergency remote instruction, which can possibly cause improvised and insufficient solutions to accommodate a new modality of learning as an alternative to classroom learning [16]. In addition, a well-thought integration of technology can potentially create more interactive experience for students in planned online learning whereas possible problems in technology may result in less interaction and worse student engagement in emergency remote instruction [16, 19]. Planned online learning gives faculty staff and teachers sufficient time to be trained in the technology that will be used for online teaching/learning as well as in online teaching pedagogy. In emergency remote instruction, both the teachers and students must undergo a fast adaptation of the technology required during online courses, which can result in several stressful moments during online learning [15–17]. Courses in planned online learning are mostly designed according to online learning/teaching requirements while courses which are forced into emergency remote instruction are generally designed for conventional classroom learning [15, 16, 18]. Emergency remote instruction is mostly delivered as synchronous while planned online learning is much more likely to be asynchronous [16]. In a planned online course, the policies and directions are commonly clearly stated for both teachers and students before the online learning takes place. However, there is always a risk that these policies cannot be provided sufficiently in emergency remote instruction [16, 17]. In planned online learning, both teachers and students have enough support for potential technology problems during online learning activities. However, the resources and support to help the teachers and students to overcome technology problems can be more limited in emergency remote instruction [15, 16].

The review of the current literature on online learning and emergency remote instruction demonstrates the importance of the current study. The main research questions we aimed to address were what teachers’ perceptions of online seminars during the COVID-19 pandemic are and how we can improve online seminars that were designed as an emergency remote instruction. We believe that the results of the present study can guide teachers in university education to make online seminars more effective in the future, especially in emergency situations like during a pandemic.

## Materials and Methods

In the current study, an anonymous questionnaire that contained 11 questions (5 close-ended questions, 5 open-ended questions and a general course evaluation question) and took approximately 20 min to complete was used to examine teachers’ reflections and perceptions to online seminars during the COVID-19 pandemic (Appendix). In total, 14 teachers, of which 9 were senior teachers and 5 were PhD students, at the Department of Pharmacology, University of Gothenburg, Sweden, filled out the questionnaire. All teachers that received the survey responded. Each teacher was assigned to teach at one or more online seminars in four different courses in pharmacology given by the department. The four courses were similar in nature, teaching pharmacology in a broad sense at a basic level to medical or pharmacy students. The teachers were tasked to guide students through the online seminars, acting both as moderators and participating in active teaching of the topic of each respective seminar. Senior teachers were defined as the teachers who had more than 10 years of teaching experience, while PhD students had briefer teaching experience (i.e. 2–5 years). All online seminar teachers were informed about the study, and permission to report the results of the survey was obtained from each teacher. There was no risk that the comments from online seminar teachers could be identifiable or traced back to the participant since the teachers answered the questions in the survey using an online platform (www.freeonlinesurveys.com). It was only possible to see if the origin of the comment was from a senior teacher or a PhD student. The teachers who answered the survey could only see a summary of the overall results, not any result for individual participants. Zoom was the online platform used during online seminars. It was decided to use Zoom since both the teachers and students had most previous experience with Zoom. In addition, Zoom was assumed to be perceived as easy to use, and there are many functions in Zoom, such as chat function and break-out rooms, that help teachers to perform effective online seminars.

The PhD students’ and senior teachers’ answers to questions 1–5 were compared statistically to quantitatively investigate potential differences between online seminar teachers in terms of previous teaching experience. Statistical calculations were performed using GraphPad Prism version 9.0.2 (GraphPad Software Inc., San Diego, USA). The Mann–Whitney *U* test was used for statistical comparisons. Statistical significance was considered for *p* values < 0.05. The total number of comments that were analysed for each question regarding questions 6–10 in the questionnaire was 13 (8 comments from senior teachers and 5 comments from PhD students, for each question). The results of questions 6–10 were presented on the basis of a qualitative inductive method with a thematic content analysis that made it possible to evaluate the teachers' perceptions to online seminars. The six steps developed by Braun and Clarke (familiarization, coding, generating themes, reviewing themes, defining/naming themes and writing up) were followed for analysis of teachers’ answers to questions 6–10 [20]. By using thematic content analysis, the seminar teachers’ opinions and experiences were examined based on the qualitative data in the questionnaire. An inductive method was used to develop a unique framework based on what the qualitative data showed in the survey. First, the data from the survey were examined to identify common themes that came up several times. Initially, a thorough overview was made of all the answers from the teachers to get acquainted with them (familiarization). Thereafter, some parts of the answers that were relevant or potentially interesting (codes) were chosen to get a summary of the most important points in the data (coding). Different themes were created using these codes (generating themes). Next, the data were checked again, and a comparison was made to ensure that nothing was overlooked (reviewing themes). Lastly, after obtaining a final list of themes, a last round of definitions was performed (defining and naming themes), and the analysis was carried out (writing up).

## Results

### Questions 1–5

**Fig. 1:**
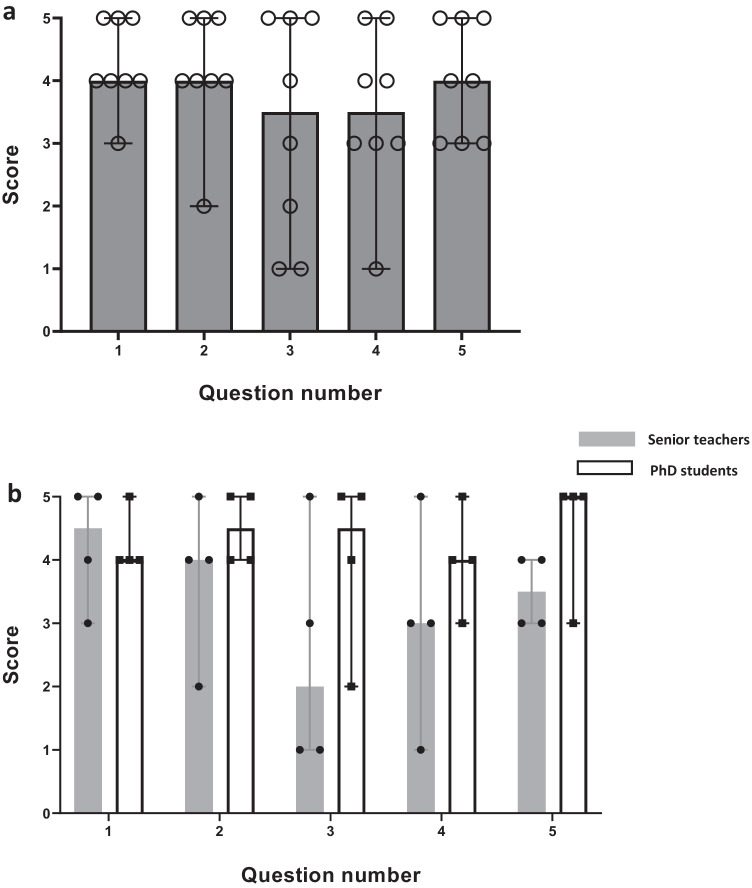
Scores given by online seminar teachers (senior teachers and PhD students) to questions 1–5 in the questionnaire (**a**) and comparison of senior teachers’ and PhD students’ scores (**b**). Online seminar teachers were tasked to choose the most appropriate answer/score (between 1 and 5: 1, I do not agree at all; 2, I do not agree; 3, Uncertain; 4, I agree; and 5, I absolutely agree) to questions 1–5. Data are presented as medians with range. There was no significant difference between senior teachers and PhD students in terms of the scores given to questions 1–5 in the questionnaire (*p* > 0.05, Mann–Whitney *U*)

Figure 1 shows the answers/scores given by online seminar teachers (senior teachers and PhD students) to questions 1–5 in the questionnaire. There were no significant differences between senior teachers and PhD students in regard to the scores given by the teachers to questions 1–5 in the questionnaire (question 1, *p* > 0.999; question 2, *p* = 0.657; question 3, *p* = 0.314; question 4, *p* = 0.429; question 5, *p* = 0.229; Fig. 1b).

### Questions 6–10

#### Less Interaction

Most seminar teachers, especially senior teachers (7 senior teachers and 2 PhD students), mentioned that the interaction between students was diminished during online seminars as compared to in person seminars:

“I feel that the interaction between students has been much worse compared to what the seminars looked like before the pandemic. They have not discussed to the same extent, only answered the questions”.

#### Poor Group Dynamics

It appears that discussion quality and group dynamics were worse due to impaired interaction between students (4 senior teachers and 2 PhD students mentioned that):

“I feel that the group dynamics got worse due to partly technical problems but also because some students interrupted the others during their presentations”.

“I couldn’t maintain the same group dynamic. It was difficult to understand if there was someone who couldn’t keep up”.

#### Using Different Functions in Zoom

The questionnaire showed that three of five PhD students thought that the interaction/communication between students worked well during online seminars, even though they did not receive as many questions from students. This might be due to the effective use of various functions such as chat function and break-out rooms in Zoom. Both teachers’ and students’ computer skills may also impact how effective the communication was during online seminars. It seems that it is also important to explain to the students that they should collaborate during online seminars to improve the communication/interaction, more important than during in person seminars:

“It has worked well. I have created breakout rooms, then I have been able to go between these”.

“Excellent, but difficult to use chat function as teachers do not always see this”.

The survey did not show any differences between PhD students and senior teachers in terms of technical challenges during online seminars. Only one senior teacher mentioned the need for a possible demonstration of Zoom prior to teaching:

“I’m not very good at technical stuff and might have wanted a zoom review before online seminars. I had for instance problems dividing students into break-out rooms”.

#### Attention and Interest

Teachers’ answers to the questions in the questionnaire showed that a common problem during online seminars was to get students involved and that some students became less involved in online seminars, which probably led to difficulties in having fruitful discussions. Most online seminar teachers (6 senior teachers and 3 PhD students) have complained that some students did not show enough interest in the topic discussed, which might be due to poorer communication between students during online seminars compared to in person seminars. However, it is not certain whether the online format was the only reason for impaired communication between students at online seminars:

“It is difficult to get students involved. However, I have also experienced that at in person seminars. So, I’m not sure if there is any difference really”.

“It is more challenging to do online seminars because students may be tired, out of focus, sleepy or just poorly motivated compared to in person seminars”.

#### Time saving and Flexibility

According to almost all seminar teachers (8 senior teachers and 4 PhD students), the obvious advantage of online seminars was the time saved by not having to go to seminar halls. Another advantage pointed out by most teachers (7 senior teachers and 3 PhD students) was that online seminars were more flexible compared to in person seminars because they were easier to coordinate. Logistical benefits have also been mentioned by some teachers:

“One of the advantages is that you do not have to go to a specific place”.

“Easier to change the time of online seminars”.

“Convenient for these students who often have long journeys and families at home. Easy to coordinate and make any last-minute changes”.

#### Dare to Ask

It seems that some students who usually do not dare to ask questions at in person seminars have participated more actively in online seminars (3 senior teachers & 2 PhD students mentioned that):

“I think those who are shy dare to ask more questions”.

Using the chat function can encourage students to ask more questions at online seminars (3 senior teachers and 3 PhD students mentioned that):

“Students tend to ask more questions through the chat feature, which is not available during seminars on in person. This can make it easier to resolve simple doubts faster”.

#### Monologue Discussion

It seems that some teachers had to dominate the discussion after a while during online seminars (4 senior teachers and 2 PhD students mentioned that), which could be due to poor group dynamics and less communication between students and teachers:

“As a teacher, it is difficult to assess whether the student is reading directly from the book and the discussion risks being changed to a monologue”.

#### Body Language

Body language is an important part of communication and lack of body language during online seminars could be a reason for impaired communication/interaction between students and teachers (2 senior teachers and 2 PhD students mentioned the importance of body language):

“Human contact is declining, and it is more difficult to know whether the students follow the discussion”.

“Communication gets worse without body language”.

#### Poor Internet Connection and Sound Quality

According to the survey, most seminar teachers have not had any major technical challenges during online seminars (6 senior teachers and 4 PhD students mentioned that). Five senior teachers and 3 PhD students mentioned that they had minor technical problems such as connection problems and poor sound quality:

“Zoom and Teams usually worked well. Only minor technical problems have been experienced”.

“The Internet shuts down sometimes. Poor sound quality”.

#### Motivate the Students

Seminar teachers came up with various suggestions to improve online seminars in the future. The most prominent among them was to motivate students to be more active during online seminars and thereby improve the group dynamics (5 senior teachers and 2 PhD students mentioned that):

“Try to motivate the students to be more active in the discussions”.

“New ways of teaching that are more interactive than just a question & answer seminar. For example, discussions after the online seminar with pre-recorded videos, interactive quiz”.

#### Smaller Groups

Three senior teachers and 3 PhD students suggested having even smaller groups by using breakout rooms. The main reasons for this were to improve group dynamics and have more interactive discussions:

“Maybe even smaller groups? Break-out rooms with 3 and 3 so that they have less [people] to interact with”.

#### Question Time

A PhD student mentioned the importance of the chat function and additional time for possible questions at the end of the seminar to improve online seminars in the future:

“Better chat function. Add time for possible questions at the end”.

#### Clear Guidelines

Some teachers (3 senior teachers and 1 PhD student) mentioned the need for clear guidelines during online seminars:

“Clear guidelines for students and teachers”.

#### Remove Online Seminars

It seems that two senior teachers became annoyed with online seminars and would prefer to do seminars on in person:

“Remove them”.

“That they are only used in emergencies = in connection with a pandemic or similar. Otherwise, I definitely prefer in person seminars”

Table 1 shows the thematic areas identified related to questions 6–10 in the questionnaire, a consistent number of sample comments, the total number of comments in the themed area, total number of comments from senior teachers and the total number of comments from PhD students.

**Table 1:** Results related to questionnaire questions 6–10 that constitute the thematic review

	**Number of sample comments**	**The total number of comments**	**Comments from senior teachers**	**Comments from PhD students**
**Less interaction**	1	9	7	2
**Poor group dynamics**	2	6	4	2
**Using different functions in Zoom**	3	11	7	4
**Attention and interest**	2	9	6	3
**Time saving and flexibility**	3	12	8	4
**Dare to ask**	2	5	3	2
**Monologue discussion**	1	6	4	2
**Body language**	2	4	2	2
**Poor internet connection and sound quality**	2	10	6	4
**Motivate the students**	2	7	5	2
**Smaller groups**	1	6	3	3
**Question time**	1	1	0	1
**Clear guidelines**	1	4	3	1
**Remove online seminars**	2	2	2	0

Four teachers (29%), of which three senior teachers and one PhD student, evaluated the online seminars much worse in relation to in person seminars while only one PhD student (7%) thought that online seminars were much better compared to in person seminars. Four teachers (29%), of which two senior teachers and two PhD students, meant that there was no difference between online seminars and in person seminars. Five (36%) teachers, of which four senior teachers and one PhD student, evaluated the online seminars as slightly worse, in comparison to in person seminars. PhD students and senior teachers mentioned similar pros and cons of online seminars.

## Discussion

This study showed that the interaction/communication and group dynamics became worse at online seminars compared to in person seminars, even though 3 PhD students believed the opposite. A common problem during online seminars was getting students involved in the discussion. According to the teachers, the main advantages of online seminars were time saving and flexibility. The current study also showed that some students who usually didn’t dare to ask at in person seminars asked more questions and participated more during online seminars. The main disadvantages of online seminars were reduced student interest, risk of monologue discussion and poorer communication without body language. Most of the online seminar teachers experienced minor technical problems with Internet connection and sound quality. To improve online seminars in the future, the teachers suggested to improve group dynamics, to have even smaller groups with the help of breakout rooms and better chat function and to have clear guidelines for both students and teachers. Most (7 senior teachers and 2 PhD students, 65%) teachers evaluated the quality of online seminars worse than in person seminars.

The current study showed that group dynamics, the interaction between students and student participation were worse during online seminars compared to in person seminars. These results differ from some previous studies. It has previously been shown that communication during online teaching, like online seminars, was evenly distributed and most students participated in the discussion during online teaching [1]. Two graduate schools, Ontario Institute for Studies in Education (University of Toronto, Canada) and Connected Education (associated with the New School of Social Research, New York, USA), offered postgraduate courses that included online seminars, online discussions and virtual cafes [21, 22]. Although the Internet connection was poor, students’ participation in discussions was high (85–90%), and students had high satisfaction. In addition, most of these students reported that online discussions were more social and enjoyable than face-to-face discussions. The difference between previous studies and the current study might be because in this study only the teachers’ perceptions of online seminars were examined. It is possible that the students' opinions differ from those of the teachers. It could also be due to potential differences between student characteristics and expectations, as well as teachers’ ways of discussing the subject during online seminars at different universities.

Communication/interaction between students at online seminars is generally accepted as a key factor and active student participation leads to the birth of various interesting ideas [5]. Khine et al. reported on a study that identified a lack of online discussion among 42 trainee teachers [23]. The authors stated that the students were not critical thinkers and were superficial learners because they failed to maintain interaction. Students should be encouraged to feel comfortable enough to ask questions and share information with as many others as possible at online seminars [1]. In the current study, online seminar teachers suggested to improve group dynamics and interaction between students to improve online seminars in the future. But how can we improve interaction, group dynamics and participation at online seminars?

Mason stated that the constructivist idea could be encouraged through the active participation of students in online discussions and through the changing role of the teacher into a guide [24]. As a good guide, teachers should give students additional reasons to participate. Teachers can use oral or written feedback or other methods to increase student motivation. An online seminar should have an intrinsic value for students and teachers should avoid encouraging participation only through external rewards such as grades [25]. For an online seminar to have an intrinsic value for the students, the teacher can mention the importance of the topic to be discussed at the seminar. Beaudoin examined the key factors affecting non-participation during online discussions [26]. Thirty percent of students said they were not sure what to say because the discussion was off topic, and 25% said they did not feel comfortable presenting their ideas. In our opinion, the role of the teacher is important to ensure that the discussion does not derail and to help the students so that they feel comfortable and participate in the discussion. It may also be the case that some students have more knowledge of the subject than the others in the group. If this is not realized by the teacher, it can lead to other students feeling inadequate and less capable. This in turn can negatively affect the interaction between students and overall participation in online seminars.

Previous studies showed that students would be more likely to attend online seminars if they receive feedback and advice from their classmates [25, 27]. Students can then have the chance to take on the role of a teacher, and the interaction between students and participation in the discussion can be further improved if students teach other students. It may therefore be a good idea to let students take responsibility for their own learning at online seminars to improve interaction and group dynamics. Some students may not participate due to lack of social interaction with their classmates or lack of group identity. It was previously stated that social interaction was necessary to make information exchange work during online seminars [1]. The interaction between the students can possibly be improved through social support, but it is a very challenging task for online seminar teachers. Jokes can help students feel welcome and teachers sharing personal experiences can lead to trust, which in turn promotes a creative pedagogical environment [1, 28]. However, any jokes must of course be appropriate and suitable for the current situation.

According to the teachers that participated in this study, time saving and flexibility were the most important advantages of online seminars. This is in accordance with previous research that showed that most students appreciate the flexibility, convenience and reduced costs associated with online learning [29]. In our study, another advantage of online seminars was that some students who usually did not dare to ask at in person seminars asked more questions and participated more actively in online seminars. Similarly, Maier and Warren argued that some students who are quiet and shy may have the opportunity to contribute more to the discussion during online seminars [27]. This result was not surprising since there are always some students who prefer to be quiet and just listen during in person seminars. It seems that these students who do not dare to ask questions in person may have the opportunity to be more active during online seminars.

Previous studies have shown that a particular disadvantage of online discussions is that they take place without the rich mix of speech and body language that helps to convey emotions [30, 31]. In the current study, some teachers highlighted poor communication without body language during online seminars. Body language is an important part of communication, and it is therefore not unexpected that communication during online seminars was worse due to lack of body language. However, this problem can potentially soon be solved with the help of technological development.

The current data revealed that the teachers suggested having smaller groups, a better chat function and clearer guidelines for both students and teachers to improve online seminars in the future. One way to generate smaller groups is to use breakout rooms. By using this, students would have the opportunity to experience simplified social interactions and improved information exchange. However, it may be more difficult for some teachers to have control over the discussion when having to monitor several groups. The chat function is also considered important because some students who do not dare to ask questions orally could type questions. A chat function can also play a role in creating friendship, group identity and social support. Similarly, Haythornthwaite showed that students preferred to use special tools, such as the chat function in Zoom, to ask for help that they would not otherwise ask in front of the whole group [32]. When it comes to clearer guidelines, it is a common problem that most of the teachers experience at online seminars. But it should be mentioned that the same problem can be present at in person seminars. In a previous study on the design of online seminars at the University of Linz, Austria, researchers showed that students perceived the detailed guidelines for work routines and specified requirements as very helpful in adapting their activities [33]. This is true also for online seminar teachers in the current study.

Compared to planned online learning, the current situation with emergency remote instruction could potentially have led to worse communication and group dynamics, less student participation and ineffectiveness of online seminars compared to in person seminars. Since emergency remote instruction frequently lacks a theoretical framing in guiding its design, the findings in the current study can be understood better by looking closer into the entities described within the community of inquiry framework. In an online learning/teaching environment, community of inquiry has three components: social presence, cognitive presence and teaching presence [34–36]. These three components are required to have a sufficient information exchange and collaboration between the students and teachers as well as a successful teaching/learning environment in online learning [19, 36]. Teaching presence refers to the planning and orientation of cognitive and social entities to achieve satisfactory learning/teaching outcomes in an online learning environment [19, 34, 36]. Social presence refers to the feasibility of the online environment for the students to represent themselves as individuals during online learning [19]. Cognitive presence refers to efficient knowledge building, problem-solving and critical thinking about potential problems, which can be achieved by successful communication/interaction between the students [19, 34]. In the current study, reduced student interest, risk of monologue discussion and worse interaction/communication between the students during online seminars could be due to potential deficiencies in these three components.

The current study showed that online seminar teachers experienced several challenges that can be observed in emergency situations like the COVID-19 pandemic. Ferri et al. divided the potential challenges of emergency remote instruction into three categories: technological, pedagogical and social challenges [37]. Technological challenges like Internet connection failures and poor sound quality were the most prominently reported by online seminar teachers in our study. One senior teacher also mentioned the need for a possible demonstration of Zoom prior to teaching. Regarding pedagogical challenges, the current study showed that a common problem during online seminars was to get students involved and that some students became less involved during online seminars. The online seminar teachers suggested to effectively use various functions such as chat function and break-out rooms in Zoom to overcome pedagogical challenges and maintain student interest during online seminars. Social challenges were also an important limitation for both students and teachers according to the findings in the current study. In another recent study, the authors aimed to investigate the opportunities and challenges of emergency remote instruction during the COVID-19 pandemic [37]. The authors mentioned that the most important social challenge during emergency remote instructions was lack of interaction. Our study supported this finding since most seminar teachers, especially senior teachers (7 senior teachers, 2 PhD students), mentioned that the interaction between students was diminished during online seminars as compared to in person seminars. Two senior teachers and two PhD students also mentioned the importance of body language for maintaining a successful interaction.

Although we received satisfactory answers for the topic we wanted to investigate, the present study has some limitations. The questionnaire was kept as short and clear as possible to increase the number of answers from teachers. The results could have been different if we had done interviews or had more questions in the questionnaire. The aim of this study was to examine teacher’s perceptions of online seminars, but student’s perceptions should also be examined in future studies.

That most of the seminar teachers (65%) evaluated the quality of online seminars worse than in person seminars might be due to most teachers longing for profound interactions, as previously experienced in a face-to-face environment. However, online seminars have some benefits that cannot be ignored. It may be a good idea to do online seminars in conjunction with face-to-face discussions on in person (blended-learning). This way, students and teachers can enjoy the richness of communication and social interactions in a face-to-face environment as well as the flexibility and convenience of online learning.

## Conclusion

The most common problems during online seminars, as compared to in person seminars, are worse communication and group dynamics as well as less student participation. However, online seminars have some advantages, such as being time saving and having greater flexibility. The existing functions in Zoom, such as break-out rooms and a chat function, can be used more efficiently to improve online seminars. Clear guidelines for both teachers and students can also help to improve online seminars in the future. Allowing students to take responsibility for their own learning can also promote a deeper understanding of the subject and improve interaction during online seminars. After the COVID-19 pandemic, blended-learning can be an excellent alternative to take advantage of the benefits of both a face-to-face environment and online learning.

## References

[1] Hrastinski S, Jaldemark J (2012). How and why do students of higher education participate in online seminars?. Educ Inf Technol.

[2] Jaldemark J (2008). Participation and genres of communications in online settings of higher education. Educ Inf Technol.

[3] Jonsson L, Säljö R. The online seminar as enacted practice. In: Stansfield MH, Connolly TM (eds) Institutional transformation through best practices in virtual campus development: advancing e-learning policies. Hershey: Idea Group. urn:nbn:se:du-15483. 2009; p. 38–54.

[4] Bates AW (Tony). Teaching in a digital age. Chapter 3&4. Vancouver BC: Tony Bates Associates Ltd. 2015; p. 81–167. https://opentextbc.ca/teachinginadigitalage/.

[5] Harasim L (2000). Shift happens. Online education as a new paradigm in learning. Internet High Educ.

[6] Kinshuk Dr, Chen NS. Synchronous methods and applications in e-learning. Campus-Wide Inf Syst. 2006;23(3). 10.1108/cwis.2006.16523caa.001.

[7] Jonassen DH, Land SM (2000). Theoretical foundations of learning environments.

[8] Chen G, Wang C, Ou K (2003). Using group communication to monitor Web-based group learning. J Comput Assist Learn.

[9] Hammond M (2005). A review of recent papers on online discussion in teaching and learning in higher education. Online Learn.

[10] Bernard RM, Borokhovski E, Schmid RF, Tamim RM, Abrami CP (2014). A meta-analysis of blended learning and technology use in higher education: from the general to the applied. J Comput High Educ.

[11] Bernard RM, Abrami PC, Borokhovski E, Wade CA, Tamim RM, Surkes MA, Bethel EC (2009). A meta-analysis of three types of interaction treatments in distance education. Rev Educ Res.

[12] Bernard RM, Abrami PC, Lou Y, Borokhovski E, Wade A, Wozney L (2004). How does distance education compare with classroom instruction? A meta-analysis of the empirical literature. Rev Educ Res.

[13] Means B, Toyama Y, Murphy R, Bakia M, Jones K. Evaluation of evidence-based practices in online learning: A meta-analysis and review of online learning studies. U.S. Department of Education Office of Planning, Evaluation, and Policy Development Policy and Program Studies Service. 2010. https://www2.ed.gov/rschstat/eval/tech/evidence-based-practices/finalreport.pdf. Accessed 21 Jan 2022.

[14] Sitzmann T, Kraiger K, Stewart D, Wisher R (2006). The comparative effectiveness of Web-based and classroom instruction: a meta-analysis. Pers Psychol.

[15] Hodges C, Moore S, Lockee B, Trust T, Bond A. The difference between emergency remote teaching and online learning. Educause Review. 2020. https://er.educause.edu/articles/2020/3/the-difference-between-emergency-remote-teaching-and-online-learning. Accessed 21 Jan 2022.

[16] Shisley S. Emergency remote learning compared to online learning. Learning Solutions. 2020. https://learningsolutionsmag.com/articles/emergency-remote-learning-compared-to-online-learning. Accessed 21 Jan 2022.

[17] Craig R. What students are doing is remote learning, not online learning. There’s a difference. EdSurge. 2020. https://www.edsurge.com/news/2020-04-02-what-students-are-doing-is-remote-learning-not-online-learning-there-s-a-difference. Accessed 21 Jan 2022.

[18] Manfuso LG. From emergency remote teaching to rigorous online learning. EdTech. 2020. https://edtechmagazine.com/higher/article/2020/05/emergency-remote-teaching-rigorous-online-learning-perfcon. Accessed 21 Jan 2022.

[19] Aslan A (2021). The evaluation of collaborative synchronous learning environment within the framework of interaction and community of inquiry: An experimental study. J Pedagogical Res.

[20] Braun V, Clarke V (2006). Using thematic analysis in psychology. Qual Res Psychol.

[21] Harasim L (1987). Teaching and learning online: issues in designing computer-mediated graduate courses. Can J Educ Commun.

[22] Levinson P, Harasim L (1990). Computer conferencing in the context of the evolution of media. Online education: perspectives of a new environment.

[23] Khine MS, Yeap LL, Chin Lok AT (2003). The quality of message ideas, thinking and interaction in an asynchronous CMC environment. Education Media International.

[24] Mason R. Models of online courses. Ed at a Distance. 2001;15(7). https://citeseerx.ist.psu.edu/viewdoc/download?doi=10.1.1.1068.66&rep=rep1&type=pdf. Accessed 21 Jan 2022.

[25] Groves M, O´Donoghue J (2009). Reflections of students in their use of asynchronous online seminars. J Educ Technol Soc.

[26] Beadoin M (2002). Learning or Lurking? Tracking the invisible online student. Internet High Educ.

[27] Maier P, Warren A (2000). Integrating technology in learning and teaching: a practical guide for educators.

[28] Hillman D (1999). A new method for analysing patterns of interaction. Am J Distance Educ.

[29] Graham CR, Moore MG (2013). Emerging practice and research in blended learning. Handbook of distance education.

[30] Kear K (2004). Peer learning using asynchronous discussion systems in distance education. Open Learning.

[31] R R, Harry K (1999). The impact of telecommunications. Higher education through open and distance learning: world review of distance education and open learning.

[32] Haythornthwaite C (2000). Online personal networks: size, composition and media use among distance learners. New Media Soc.

[33] Putz P, Arnold P (2001). Communities of practice: guidelines for the design of online seminars in higher education. Educ Commun Inf.

[34] Garrison DR, Anderson T, Archer W (1999). Critical inquiry in a text-based environment: computer conferencing in higher education. Internet High Educ.

[35] Garrison DR, Anderson T, Archer W (2001). Critical thinking, cognitive presence, and computer conferencing in distance education. Am J Distance Educ.

[36] Shea P, Bidjerano T (2012). Learning presence as a moderator in the community of inquiry model. Comput Educ.

[37] Ferri F, Grifoni P, Guzzo T (2020). Online learning and emergency remote teaching: opportunities and challenges in emergency situations. Societies.

